# Spatial variation of insecticide resistance in the dengue vector *Aedes aegypti* presents unique vector control challenges

**DOI:** 10.1186/s13071-016-1346-3

**Published:** 2016-02-04

**Authors:** Regan Deming, Pablo Manrique-Saide, Anuar Medina Barreiro, Edgar Ulises Koyoc Cardeña, Azael Che-Mendoza, Bryant Jones, Kelly Liebman, Lucrecia Vizcaino, Gonzalo Vazquez-Prokopec, Audrey Lenhart

**Affiliations:** Department of Environmental Sciences, Emory University, Atlanta, GA USA; Unidad Colaborativa para Bioensayos Entomológicos, Campus de Ciencias Biológicas y Agropecuarias, Universidad Autónoma de Yucatán, Mérida, Mexico; Servicios de Salud de Yucatán, Gobierno del Estado de Yucatán, Mérida, Mexico; Division of Parasitic Diseases and Malaria, Entomology Branch, Centers for Disease Control and Prevention, Center for Global Health, Atlanta, GA USA

**Keywords:** *Aedes aegypti*, Dengue, Insecticide resistance, *kdr*

## Abstract

**Background:**

Dengue is a major public health problem in Mexico, where the use of chemical insecticides to control the principal dengue vector, *Aedes aegypti*, is widespread. Resistance to insecticides has been reported in multiple sites, and the frequency of *kdr* mutations associated with pyrethroid resistance has increased rapidly in recent years. In the present study, we characterized patterns of insecticide resistance in *Ae. aegypti* populations in five small towns surrounding the city of Merida, Mexico.

**Methods:**

A cross-sectional, entomological survey was performed between June and August 2013 in 250 houses in each of the five towns. Indoor resting adult mosquitoes were collected in all houses and four ovitraps were placed in each study block. CDC bottle bioassays were conducted using F_0_-F_2_ individuals reared from the ovitraps and *kdr* allele (Ile1016 and Cys1534) frequencies were determined.

**Results:**

High, but varying, levels of resistance to chorpyrifos-ethyl was detected in all study towns, complete susceptibility to bendiocarb in all except one town, and variations in resistance to deltamethrin between towns, ranging from 63–88 % mortality. Significant associations were detected between deltamethrin resistance and the presence of both *kdr* alleles. Phenotypic resistance was highly predictive of the presence of both alleles, however, not all mosquitoes containing a mutant allele were phenotypically resistant. An analysis of genotypic differentiation (exact G test) between the five towns based on the adult female *Ae. aegypti* collected from inside houses showed highly significant differences (*p* < 0.0001) between genotypes for both loci. When this was further analyzed to look for fine scale differences at the block level within towns, genotypic differentiation was significant for both loci in San Lorenzo (Ile1016, *p* = 0.018 and Cys1534, *p* = 0.007) and for Ile1016 in Acanceh (*p* = 0.013) and Conkal (*p* = 0.031).

**Conclusions:**

The results from this study suggest that 3 years after switching chemical groups, deltamethrin resistance and a high frequency of *kdr* alleles persisted in *Ae. aegypti* populations. The spatial variation that was detected in both resistance phenotypes and genotypes has practical implications, both for vector control operations as well as insecticide resistance management strategies.

## Background

Dengue is the most important and widespread mosquito-borne viral infection of humans in the world [1]. An estimated 390 million cases of dengue virus (DENV) infection occur per year throughout the tropical and subtropical world. It is estimated that up to 55 % of the world’s population is at risk of infection in 128 countries, where 824 million people live in urban environments [1]. In the last 20 years, dengue epidemics have increased in both number and magnitude, due to a range expansion of the *Aedes aegypti* mosquito, the primary vector of dengue viruses, as well as increased trends in urbanization and global travel and weakened public health infrastructure [2, 3].

In the absence of effective therapeutic medications and vaccines, *Ae. aegypti* vector control is presently the only approach for preventing and controlling dengue virus transmission [4, 5]. Vector control strategies rely heavily on the application of chemical insecticides which target immature mosquitoes in their development sites and adult mosquitoes which are often targeted through the use of ultra-low-volume (ULV) and indoor space spraying [5, 6]. Due to the daytime biting behavior of female *Ae.aegypti*, the use of bednets as a protective barrier is not generally recommended, although the presence of insecticide treated bednets in houses has been associated with lowered dengue vector infestation indices [7]. Other insecticide-treated materials such as window and door curtains and screens and water storage container covers have also been shown to be effective at reducing household-level *Ae. aegypti* infestations [8–15], but the impact of such entomological reductions on the risk of dengue virus transmission remains to be determined [16].

As the importance of dengue as a public health problem has increased globally, insecticide-based vector control interventions have been widely employed. The heavy reliance on chemical insecticides to control dengue vectors has led to the development of resistance to many of the insecticides most commonly applied. In the Americas alone, resistance to multiple classes of insecticides has been widely reported in *Ae. aegypti* [17–23]. This is cause for alarm to those involved in dengue prevention and control, as the growing prevalence of resistance could threaten vector control efficacy [21, 23–25].

Resistance to insecticides can be caused by a variety of physiological changes within a mosquito, including structural alterations at the target site of the insecticide, increased activity of enzymes associated with insecticide detoxification, and changes to the mosquito cuticle. Non-synonymous mutations in the voltage-gated sodium channel transmembrane protein (*para*) can result in ‘knockdown resistance’ (*kdr*), in which the binding of DDT and pyrethroid insecticides are reduced at this target site [26–29]. There are several point mutations known to confer *kdr*-type insecticide resistance in *Ae. aegypti*. In the Americas, substitutions at codons 1016 (V1016I) and 1534 (F1534C) on domains II and III of the voltage gated sodium channel are strongly associated with DDT and pyrethroid resistance in *Ae. aegypti* [27, 30, 31].

Pyrethroids have become the most frequently used public health insecticides globally due to their low cost and low toxicity to mammals, in addition to their high residual power [32]. It is of considerable concern when *kdr* is found in wild populations of vector mosquitoes, given there are few suitable alternatives to pyrethroid insecticides approved for public health use. Previous research has demonstrated that *kdr* mutations arose and spread rapidly in Mexican *Ae. aegypti* populations. Between 2007 and 2009, Siller et al. [33] reported an increase in the frequency of the Ile1016 allele in several localities within the state of Veracruz, Mexico. Similar findings by Ponce Garcia et al. [34] showed over the course of 14 years, several Mexican states, including Veracruz and Yucatan, experienced a significant increase in the frequency of the Ile1016 allele. In the city of Merida, in Yucatan State, Ile1016 had not been detected in 1999, yet by 2007, it was occurring at frequencies as high as 54 %. A more recent study in the state of Guerrero, in southern Mexico, found Ile1016 at a frequency of 80 %,with all mosquitoes also containing the Cys1534 allele [35]. The F1534C substitution was initially reported in *Ae. aegypti* mosquitoes in Asia and first detected in North America in the Cayman Islands [30]. The detection of the Cys1534 allele in Guerrero was the first report of that particular *kdr* mutation in Mexico [35].

Dengue outbreaks have been reported in Mexico since 1979, and all four DENV serotypes have been reported in Mexico [36]. It is well documented that as viral serotypes are displaced by the introduction of new serotypes, the incidence of severe dengue increases [37, 38]. Yucatan State experienced a similar trend with the incidence of severe dengue increasing each time a new serotype was introduced [12]. The city of Merida is the capital and main urban center of Yucatan State, and dengue transmission in Merida is hyper-endemic. In recent decades, co-circulation of multiple serotypes and a high abundance of *Ae.aegypti* has increased the risk of severe dengue for the population living in and around the city center [12].

From 1998 to 2009, pyrethroids were the primary insecticides used for adult mosquito control in Mexico, and deltamethrin was the primary insecticide used for indoor space spraying when a dengue case was reported. In 2010, reports of high levels of pyrethroid resistance and a high frequency of the Ile1016 allele in Mexico [34, 35] prompted vector control authorities to modify their strategy. From 2010 up until when collections for this study were conducted in 2013, carbamates (bendiocarb, and later propoxur) had been predominantly used for indoor space spraying, although the pyrethroids bifenthrin and sumithrin continued to be used at a reduced level.

While the intensity of insecticide use for dengue vector control in the city of Merida is relatively consistent, the surrounding communities have experienced more sporadic insecticide applications. Little entomological data, including resistance data, are available from these surrounding towns. Previous research [34] has suggested a high degree of heterogeneity in insecticide resistance frequency across the Yucatan Peninsula, yet little is known about these patterns of resistance at a finer spatial scale. Spatial heterogeneity of insecticide resistance could have important implications for vector control efficacy, particularly when vector control strategies are designed to be applied across a large geographical area. The development of scientifically sound vector control and insecticide resistance management strategies for *Ae. aegypti* depends on understanding the patterns and drivers of spatial heterogeneity in insecticide resistance. In the present study, we characterized patterns of insecticide resistance in *Ae. aegypti* populations within five towns surrounding the city of Merida, Mexico, to gain a greater understanding of how resistance may be distributed at a fine geographical scale.

## Methods

### Study area

**Fig. 1:**
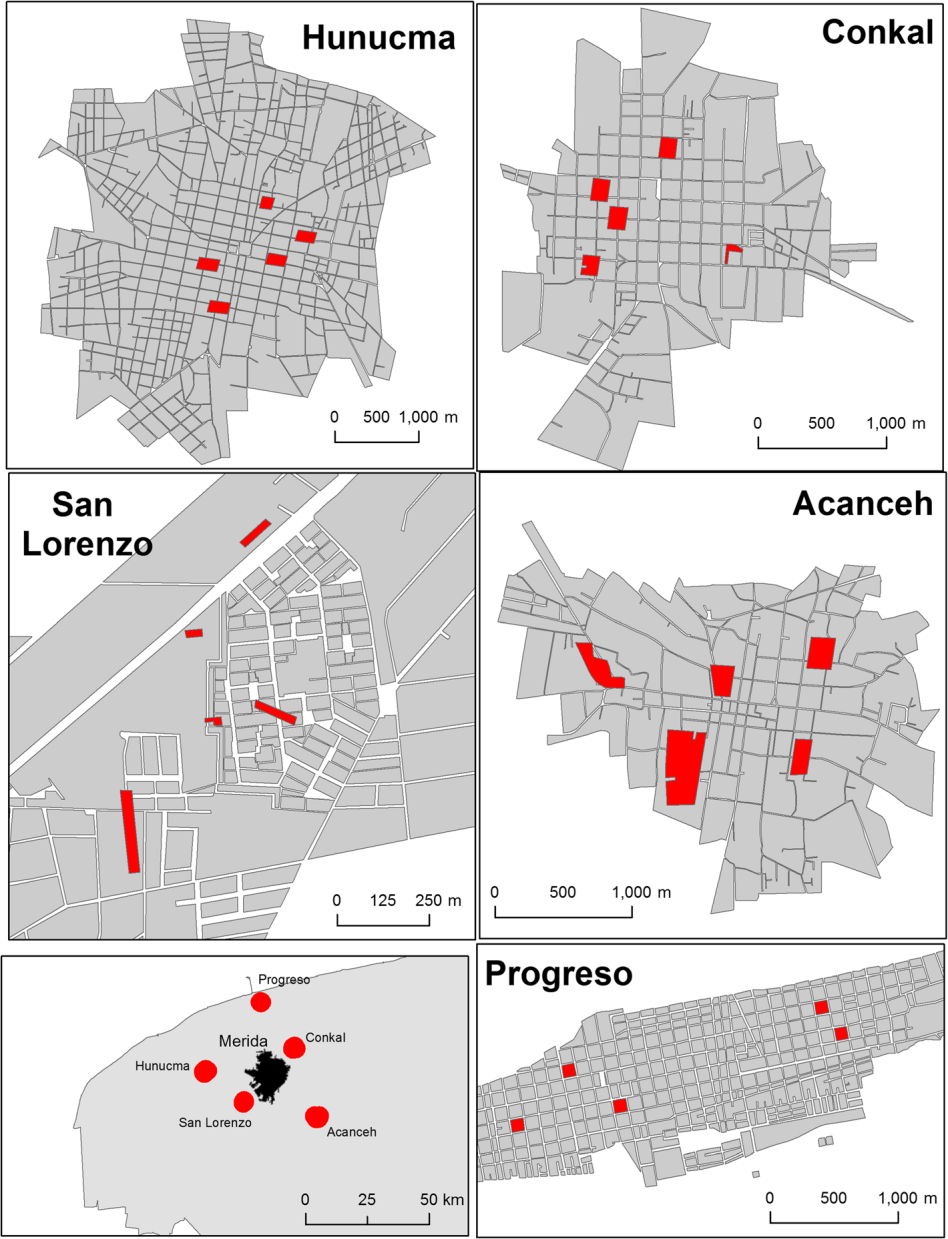
Maps showing the study areas. Location of Merida, Yucatan, Mexico and the five study towns (*lower left panel*), with each block sampled in each town highlighted

This study was conducted in the state of Yucatán in southern Mexico, and included five towns located on the periphery of the state’s capital, Merida (population ~1 million). Merida is located in a subtropical environment with mean temperatures ranging from 29 °C in December to 34 °C in July. The rainy season occurs from May to October, which overlaps with the peak dengue transmission season between July and October, although cases occur year-round [39]. Dengue is highly endemic throughout the Yucatan peninsula, and the vector control strategies used at the time of this study included ultra-low volume (ULV) spraying with the organophosphate insecticide chlorpyrifos-ethyl, indoor space spraying with the pyrethroid deltamethrin or the carbamate bendiocarb, and the application of the organophosphate larvicide temephos for breeding site control (Che-Mendoza, Secretaria de Salud de Yucatan, personal communication). Surrounding Merida are small, densely populated, satellite towns that are normally connected to Merida by a single road. The five towns selected for this study were San Lorenzo, Acanceh, Progreso, Hunucma and Conkal. All towns were located 15–35 km from Merida’s city center and at least 20 km from one another (Fig. 1). Each town has its own municipal jurisdiction, including environmental management entities who engage in breeding site control activities in addition to the vector control interventions conducted by the state-level vector control authorities.

### Study design

A cross-sectional, entomological survey was performed between June and August 2013 in 250 houses across the five satellite towns. A spatially random selection of houses nested in blocks was performed to representatively capture the geography of each town. Briefly, the location of all the urban blocks of each town was digitized using a Geographic Information System (ArcGIS 10.1, ESRI, Redlands CA). This block-level map was then used to group the blocks into 90° sectors equivalent to the cardinal directions. One block was randomly selected from each one of the sectors (total four blocks) and one additional block was randomly selected from the most central blocks in the town (Fig. 1). On each sampling block, ten houses were randomly selected to perform the entomological collections.

### Entomological collections

Indoor adult mosquito collections were performed between 8:00 am and 2:00 pm by five two-person teams using Prokopack aspirators [40]*.* Collections were limited to a maximum of 10–15 min per team per household. Each premise was additionally checked for the presence of *Ae. aegypti* breeding sites. All collected adult mosquitoes were killed by freezing and visually identified to species. All *Ae. aegypti* females were subsequently desiccated and stored at -20 °C for future molecular analysis.

*Ae. aegypti* ovitraps [41] consisting of 5 l dark buckets were placed in the houses located closest to the corners of each block (four traps per block) to collect *Ae. aegypti* eggs. Ovitrap fabric was checked weekly for a maximum of 3 weeks, and the fabric containing *Ae. aegypti* eggs was dried and stored in sealed plastic bags until rearing. Eggs were subsequently reared under insectary conditions to provide material for the insecticide resistance bioassays.

### Insecticide bioassays

Eggs collected from the ovitraps in each town were pooled to generate a single, geographically diverse colony for each town. The eggs were hatched and reared to adulthood under constant temperature and humidity conditions at the CDC insectaries (Atlanta, USA). Adult females emerging from the five *Ae. aegypti* colonies were evaluated for resistance to deltamethrin, bendiocarb and chlorpyrifos-ethyl using the CDC bottle bioassay protocol [42]. Mortality at the CDC-recommended diagnostic doses (DD; deltamethrin: 10 μg/bottle, bendiocarb: 12.5 μg/bottle, chlorpyrifos: 50 μg/bottle) was recorded at the diagnostic time (DT) of 30 min. [42] For each town, 3-4 replicates were conducted for each insecticide, and approximately 20 female *Ae. aegypti* were used per bottle (mean = 20.8, standard deviation = 1.7).

Tests were conducted on F_0_-F_2_*Ae. aegypti*. Mosquitoes were classified as phenotypically resistant or susceptible to each insecticide based on their knockdown status at the DT. Individuals were considered to be knocked down if they were no longer able to stand at the diagnostic time. Both resistant and susceptible individuals from the bioassays were subsequently *kdr*-genotyped for Ile1016 and Cys1534.

### Molecular assays

Genomic DNA was extracted from a leg or other body part from each individual adult female mosquito in a solution of 45 μl of H_2_O and 5 μl of Promega Taq DNA Polymerase10x Buffer with MgCl_2_ (Madison, WI) in a 96 well PCR plate. Samples were incubated at 95 °C in a Bio-Rad iCycler™ thermal cycler for 15 min.

Allele-specific PCR was carried out in a Bio-Rad CFX96 Real-Time System C1000 thermal cycler to determine genotype at both loci through analysis of the PCR product melting curves. The PCR reaction to detect the Ile1016 allele was based on the methodology described by Saavedra-Rodriguez et al*.* [27] and consisted of 4 μl of iQ™ SYBR® Green Supermix (Bio-Rad 170-8880), 2 μl of each of the Val1016f, Ile1016f and Ile1016r primers, and1 μl of DNA template. The PCR reaction to detect the Cys1534 allele was based on the methodology described by Yanola et al*.* [43] and consisted of 7.67 μl of iQ™ SYBR® Green Supermix (Bio-Rad 170-8880),1 μl each of the Phe1534 + f and Phe1534 + r primers and 0.33 μl of the Cys1534 + f primers, and 1 μl of DNA template [43]. Results were read using Precision Melt Analysis Software™. For the 1016I mutation, a melting peak at 79 °C corresponds to isoleucine and a melting peak at 85 °C corresponds to valine (wild type). For the 1534C mutation, a peak at 85 °C corresponds to cysteine and a peak at 80 °C corresponds to phenylalanine (wild type). Genomic DNA from the Rockefeller *Ae. aegypti* strain was used as a susceptible (wild-type) control. DNA from previously genotyped individuals was used for positive controls for both *kdr* mutations.

### Data analysis

Per CDC bottle bioassay guidelines, populations were classified as resistant or susceptible using the updated WHO guidelines [44]: 98–100 % mortality indicates susceptibility, 90–97 % mortality suggests resistance may be developing, and mortality less than 90 % indicates resistance.

The allele frequencies for Ile1016 and Cys1534 were calculated using the equation:

$$ \frac{\mathrm{n}\ \mathrm{heterozygotes} + 2\left(\mathrm{n}\ \mathrm{homozygotes}\right)}{2\left(\mathrm{total}\ \mathrm{n}\ \mathrm{mosquitoes}\ \mathrm{analyzed}\right)} $$

The 95 % confidence interval (CI95) around the frequency of each of the alleles was calculated using a Wald interval [34]. Fisher’s exact tests were performed in SAS 9.3 to test the association between genotype and resistance phenotype. Analysis of linkage disequilibrium was also conducted between sites 1016 and 1534 using Genepop version 4.2 [45].

A genotypic differentiation analysis was performed using Genepop version 4.2 on the adult female *Ae. aegypti* collected from inside the houses to assess the significance of genotype differences between towns (exact G test) and for genotype differences between blocks within each town [45].

## Results

A total of 545 adult female *Ae. aegypti* were collected from inside the houses across the five towns (Table 1).

**Table 1:** Adult female *Ae. aegypti* collected from houses

Town	No. female *Ae. aegypti* collected	Median	Q1	Q2	Q3	No. with PCR result for one or both kdr alleles
San Lorenzo	155	38	13	38	46	141
Acanceh	100	16	12	16	32	91
Progreso	61	11	8	11	12	60
Hunucma	124	20	19	20	31	117
Conkal	105	15	8	15	31	103
Total	545					512

Median and quartile values of the number of mosquitoes collected per block are shown. Also shown are the number of house-collected mosquitoes that successfully amplified in the molecular assays to detect Ile1016 and Cys1534

### Insecticide bioassays

**Fig. 2:**
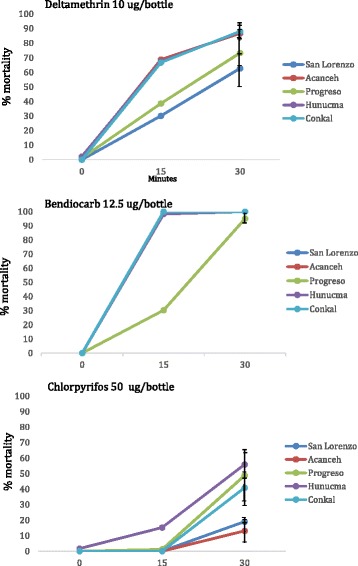
Bioassay results. Insecticide resistance bioassay results (± SE) for female *Ae. aegypti* from each of the five towns exposed to diagnostic doses of deltamethrin (*top*), bendiocarb (*middle*), and chlorpyrifos-ethyl (*bottom*) using the CDC bottle bioassay. The diagnostic time for all three insecticides was 30 min

CDC bottle bioassays were performed in which 402 female *Ae. aegypti* were tested for resistance to chlorpyrifos-ethyl, 359 were tested for resistance to bendiocarb, and 429 were tested for resistance to deltamethrin. Resistance to chlorpyrifos-ethyl was observed in all five communities at differing levels, ranging in mortality from 13.8 % (SE ± 8.0) in Acanceh to 57.2 % (SE ± 8.7) mortality in Hunucma. The development of resistance to bendiocarb was only detected in one of the five communities, Progreso (95 % mortality, SE ±3.5), while all other communities demonstrated complete susceptibility (100 % mortality). Resistance to deltamethrin also varied between the communities; San Lorenzo showed the lowest mortality rate at 62.7 % (SE ± 11.6) while Conkal showed the highest mortality rate at 88.1 % (SE ± 5.7) (Fig. 2).

### Kdr genotyping

PCR to detect Ile1016 and Cys1534 was performed on 422/429 mosquitoes tested for deltamethrin resistance in the CDC bottle bioassay. Of the 545 adult female *Ae. aegypti* collected from the houses, 512 were tested for Ile1016 and 507 were tested for Cys1534. (Tables 2 and 3). From all mosquitoes genotyped for the Ile1016 allele (*n* = 934), 20.0 % (*n* = 187) were wild-type (susceptible) homozygotes (SS), 36.8 % (*n* = 344) were heterozygotes (SR) and 43.1 % (*n* = 403) were homozygous resistant (RR). From all mosquitoes genotyped for the Cys1534 allele (*n* = 929), 10.3 % (*n* = 96) were SS, 30.5 % (*n* = 283) SR and 59.2 % (*n* = 550) RR.

**Table 2:** Summary of data relating deltamethrin resistance phenotype to *kdr* genotype per town

			V1016I					F1534C					Double homozygotes	
Town	Deltamethrin Phenotype	n	V/V	V/I	I/I	Freq. I	*p*	F/F	F/C	C/C	Freq. C	*p*	V/V & F/F	I/I & C/C
San Lorenzo	susceptible	52	0	38	14	0.63		0	8	44	0.92		0	13
	resistant	31	1	4	26	0.9	<.0001	1	0	30	0.97	0.012	1	26
	Total	83	1	42	40	0.74		1	8	74	0.94		1	39
Acanceh	susceptible	72	19	45	8	0.42		7	40	25	0.63		7	8
	resistant	10	0	0	10	1.0	<.0001	0	0	10	1.0	<.0001	0	10
	Total	82	19	45	18	0.49		7	40	35	0.67		7	18
Progreso	susceptible	66	10	27	29	0.64		0	8	58	0.94		0	29
	resistant	24	0	7	17	0.85	0.0021	0	0	24	1.0	0.074	0	17
	Total	90	10	34	46	0.7		0	8	82	0.96		0	46
Hunucma	susceptible	77	18	54	5	0.42		8	56	13	0.53		8	5
	resistant	11	0	2	9	0.91	<.0001	0	0	11	1.0	<.0001	0	9
	Total	88	18	56	14	0.48		8	56	24	0.59		8	14
Conkal	susceptible	70	8	45	17	0.56		7	39	24	0.62		4	15
	resistant	9	0	1	8	0.94	<.0001	0	1	8	0.93	0.051	0	8
	Total	79	8	46	25	0.61		7	40	32	0.66		4	23
TOTAL	susceptible	337	55	209	73			22	151	164			19	70
	resistant	85	1	14	70			1	1	83			1	70
	Total	422	56	223	143			23	152	247			20	140

*n* = number of individuals tested for *kdr* genotype from each community; V/V and F/F are SS (homozygous susceptible); V/I and F/C are SR (heterozygotes); I/I and C/C are RR (homozygous resistant)

**Table 3:** Frequency of Ile1016 (I) and Cys1534 (C) *kdr* alleles in indoor resting adult female *Ae. aegypti*

Town	Allele	n	Freq.	95 % Confidence limits	Genotypic differentiation *p*-value	Hardy-Weinberg *p*-value
					Between blocks within the town	
San Lorenzo	I	113	0.674	±0.086	*0.002*	*<0.0001*
	C	133	0.931	±0.043	*0.0001*	*<0.0001*
Acanceh	I	73	0.708	±0.104	*0.001*	*<0.0001*
	C	68	0.69	±0.110	0.102	*<0.0001*
Progreso	I	55	0.75	±0.114	0.4	0.492
	C	57	0.915	±0.072	0.35	0.042
Hunumca	I	66	0.425	±0.119	0.565	*<0.0001*
	C	88	0.568	±0.104	0.823	0.430
Conkal	I	74	0.586	±0.112	*0.005*	*<0.0001*
	C	158	0.563	±0.104	0.092	0.0685
					Between the five towns	
*Total*	I	381	0.626	±0.049	*<0.0001*	*<0.0001*
	C	434	0.727	±0.042	*<0.0001*	*<0.0001*

From genotyping the mosquitoes used in the deltamethrin bioassays, a highly significant association between genotype at position 1016 and deltamethrin resistance was detected in all five communities: San Lorenzo, Acanceh, Hunucma, and Conkal (*p* <0.0001), Progreso (*p* =0.002). A significant association between deltamethrin resistance and genotype at position 1534 was seen in three of the five communities: San Lorenzo (*p* =0.012) and Acanceh and Hunucma (*p* <0.0001) (Table 2).

Of the mosquitoes resistant to deltamethrin, 98.8 % (*n* = 84/85) were positive for both Ile1016 and Cys1534; 82.4 % (70/85) of those were RR at position 1016 and 97.6 % (83/85) were RR at position 1534. Of the mosquitoes homozygous RR at position 1016, approximately half (49.0 %; 70/143) were resistant to deltamethrin, whereas only a third (33.6 %; 83/247) of the mosquitoes homozygous RR at position 1534 were resistant to deltamethrin. Although there were 140 mosquitoes homozygous RR for both alleles, only 50.0 % (*n* = 70/140) of these were phenotypically resistant to deltamethrin. Interestingly, 97.9 % (*n* = 140/143) of Ile1016 RR individuals were also Cys1534 RR homozygotes, but only 56.7 % (*n* = 140/247) Cys1534 RR individuals were also Ile1016 RR homozygotes. Only three of the Phe1534 SS individuals had anIle1016 allele. As reported in other studies assessing the association between these two resistant alleles [30, 46, 47], these findings found highly significant linkage disequilibrium between the two loci (*p* < 0.001).

Combining the genotype data from the mosquitoes used in the bioassays and the adult female *Ae. aegypti* collected from the houses, the overall frequency of Ile1016 across all five towns was 60.6 % (95 % CI ±10.5). However, when broken down between towns, the Ile1016 allele frequency varied: Progreso had a frequency of 72.0 % (CI ±9.7), San Lorenzo 69.6 % (CI ±9.9), Acanceh 60.5 % (CI ±10.5), Conkal 59.5 % (CI ±10.6), and Hunucma 47.8 % (CI ±10.8). The overall frequency of the Cys1534 allele across all five towns was 83.0 % (CI ±8.1) but also demonstrated variation when broken down across sites: Progreso had a frequency of 94.0 % (CI ±5.1), San Lorenzo had a frequency of 93.4 % (CI ±5.3), Acanceh 68.0 % (CI ±10.0), Conkal 59.8 % (CI ±10.6), and Hunucma 57.8 % (CI ±10.6).

To obtain a more focused ‘snapshot’ of allele frequencies across the towns, genotypes at positions 1016 and 1534 were analyzed separately for the adult female mosquitoes that were collected from the houses (Table 3). The analysis of genotypic differentiation (exact G test) between the five towns based on the adult female *Ae. aegypti* collected from inside houses showed highly significant differences (*p* < 0.0001) between genotypes for both loci. When this was further broken down to look for fine scale differences at the block level within towns, genotypic differentiation was significant for both loci in San Lorenzo (Ile1016, *p* = 0.018 and Cys1534, *p* = 0.007) and for Ile1016 in Acanceh (*p* = 0.013) and Conkal (*p* = 0.031).

## Discussion

Our results suggest variability in both resistant phenotypes and genotypes between towns at a small geographical scale, illustrating the complex and focal nature of insecticide resistance. Our results also confirmed the association between two *kdr* mutations and phenotypic resistance to deltamethrin [30], and detected significant differences in *kdr* allele frequency at multiple spatial scales.

Low mortality was observed in all populations tested for susceptibility to chlorpyrifos-ethyl, demonstrating high levels of resistance to this organophosphate insecticide across the study area. Although all populations were resistant to chlorpyrifos, a high degree of variability was evident, with Acanceh and San Lorenzo showing the greatest resistance (<20 % mortality) and the remaining towns showing between 40 and 56 % mortality. Interestingly, susceptibility to the carbamate insecticide bendiocarb was high in all communities except Progreso, where mortality was 95 % (suggesting incipient resistance). That such a high level of resistance to an organophosphate didn’t hold true with respect to a carbamate suggests that the mechanisms underlying the resistance are unlikely to be due to *ace-1* target site insensitivity, as this would normally result in cross resistance between both organophosphate and carbamate insecticides [48], nor have *ace-1* mutations been widely reported in *Ae. aegypti*.

Mortality data from the deltamethrin bioassays also showed variation across the study sites. While all populations were categorized as resistant, the most resistant populations, San Lorenzo and Progreso, had mortalities of 63 and 73 %, respectively, while the remaining towns all had mortalities between 87 and 88 %. Significant associations were detected between deltamethrin resistance phenotype and the presence of both *kdr* alleles (Ile1016 and Cys1534). Phenotypic resistance was highly predictive of the presence of both alleles, however, not all mosquitoes containing a mutant allele were phenotypically resistant. This is not entirely unexpected, as both Ile1016 and Cys1534 are largely recessive alleles [27, 30]; indeed, our data supported this as over 90 % of heterozygotes for either allele were phenotypically susceptible to deltamethrin.

The Ile1016 allele is known to be associated with resistance to type I and II pyrethroids (including deltamethrin), as well as DDT [27], while the Cys1534 allele is thought to be associated most strongly with resistance to type I pyrethroids such as permethrin, as well as DDT [30]. However, recent evidence suggests that deltamethrin exposure selects for Cys1534 more rapidly than for Ile1016 [49]. Given that not all mosquitoes that were resistant to deltamethrin in the bioassays were homozygous for the *kdr* alleles suggests that multiple resistance mechanisms are contributing to the resistant phenotypes, most likely including metabolic mechanisms arising from the overproduction of detoxifying enzymes [50]. The role of the S989P *kdr* mutation is also of potential interest, as it has recently been associated with deltamethrin resistant phenotypes in Asian populations of *Ae. aegypti* [51, 52]. Investigation into these additional mechanisms and the role detoxifying enzymes may have on resistance to other insecticides in these populations will help to further explain the resistance patterns observed.

The population-level frequencies of both alleles were estimated from the indoor resting adult *Ae. aegypti* females collected from houses. Genotypic differentiation was significant between the five communities and was further detected between blocks within the same town in San Lorenzo for both *kdr* alleles and in Acanceh and Conkal for the Ile1016 allele. These results indicate that even at a fine geographical scale, *kdr* frequencies can differ significantly. This is particularly interesting in light of population genetics studies that have shown that *Ae. aegypti* populations in the Yucatan experience free gene flow within 180 km [53]. This suggests that the observed differences in *kdr* genotype are being driven by fine-scale pressures, such as focal insecticide exposure, as was hypothesized in a recent population genetics study by Saavedra et al. [47]. This could suggest an important role in the household use of insecticides in maintaining pyrethroid pressure, even when vector control programs have switched chemical groups. Indeed, the results from this study suggest that 3 years after the vector control program ceased deltamethrin use, deltamethrin resistance and a high frequency of *kdr* alleles persisted in the *Ae. aegypti* population.

Some evidence suggests that pyrethroid resistance has an associated fitness cost resulting in reduced larval development and adult longevity [54], although conflicting data suggest that the Cys1534 allele does not confer fitness costs in *Ae. aegypti* [55]. Evidence of duplication of the sodium channel gene has recently been reported in *Ae. aegypti* [56], and such an event could maintain both wild-type and mutant alleles in the population, simultaneously conferring resistance and reducing associated fitness costs. Although we currently do not know if this duplication has occurred in *Ae. aegypti* in Mexico, this warrants further investigation, particularly when discerning why such a high frequency of pyrethroid resistance has been maintained despite a near absence of pyrethroid pressure from vector control authorities during the period leading up to our entomological collections.

While this study presents novel findings regarding the variability of insecticide resistance in space, the interpretation of these results is limited by the cross sectional nature of the study and the small number of insecticides and resistance mechanisms investigated. It will be important to further investigate the patterns and drivers of variability in these communities to assess how insecticide use and other factors can drive resistance patterns and explain fluctuations in both space and time. The observed differences in the frequency of *kdr* alleles in these populations may have occurred due to pressure from widespread applications of insecticides. A recent study conducted in Martinique found that genetic patterns in *Ae. aegypti* were driven by insecticide pressure [57]. An alternative explanation is that the alleles were introduced through the immigration of mosquitoes from elsewhere, via containers harboring eggs or adult mosquitoes through other forms of transportation [58–60]. Understanding how these factors contribute to the establishment and maintenance of resistance is key to developing effective resistance management and prevention strategies.

The variations in both resistance phenotypes and genotypes detected in this study have practical implications for vector control operations. The observation of variability between blocks within the same community suggests that blanket vector control strategies at the provincial or even municipal levels may yield mixed results at best, and further highlights the operational challenge of managing resistance in vector populations with varying resistance profiles at a fine spatial scale. Fluctuations in resistance over time will likely further complicate the landscape, highlighting the need for systematic and routine insecticide resistance surveillance.

## Conclusions

The limited number of suitable insecticides for vector control and the logistical constraints inherent in vector control programs make the small-scale tailoring of vector control interventions particularly challenging, but this is a challenge that must be considered given that insecticide resistance can vary over a small scale. The use of multiple active ingredients through rotations or mixtures has been suggested as an effective way to mitigate or stall the emergence of resistance [61–63], at least in the short term. The findings presented here suggest that large-scale blanket vector control strategies may have limited efficacy given that vector populations can vary widely in their susceptibility to insecticides over a small geographical scale. The development of evidence-based resistance management strategies with respect to dengue vectors should be a priority as dengue continues to increase as a global public health problem.
